# Prognostic significance of early platelet dynamics in *Staphylococcus aureus* bacteremia

**DOI:** 10.1186/s12879-023-08046-w

**Published:** 2023-02-07

**Authors:** Rachid Douglas-Louis, Mimi Lou, Brian Lee, Emi Minejima, Juliane Bubeck-Wardenburg, Annie Wong-Beringer

**Affiliations:** 1 Department of Clinical Pharmacy, University of Southern California (USC) Alfred E. Mann School of Pharmacy, 1985 Zonal Ave, Los Angeles, CA 90089 USA; 2 Los Angeles County and USC Medical Center, Los Angeles, CA USA; 3 Department of Pediatrics and Division of Pediatric Critical Care Medicine, Washington University School of Medicine, St. Louis, MO USA; 4 Department of Pharmacy, Huntington Memorial Hospital, Pasadena, CA USA

**Keywords:** *Staphylococcus aureus*, Bacteremia, Thrombocytopenia, Mortality

## Abstract

**Background:**

Platelets are recognized as key immune effectors, but they are targets of bacterial virulence factors. In the present study, we aimed to examine the relationship between early platelet dynamics and the outcome of *Staphylococcus aureus* bacteremia (SAB).

**Method:**

Electronic medical records of adult patients hospitalized for SAB between July 2012 and November 2020 were retrospectively reviewed for relevant demographic, laboratory, and clinical data. The outcome endpoints were mortality and microbial persistence.

**Results:**

Among the 811 patients evaluated, 29% experienced thrombocytopenia on Day 1. Platelet count nadir occurred on Days 2–3 following SAB onset, and Day 4 was a determining point of platelet count trajectory and mortality. Mortality risk was 6% or less for those with normal platelet count by Day 4 regardless of whether they experienced thrombocytopenia on Day 1, but the risk increased to 16–21% for those who experienced thrombocytopenia on Day 4 regardless of whether they had normal platelet count on Day 1 or sustained thrombocytopenia. The duration of bacteremia was prolonged by one day (median 3 d vs. 2 d) for those with sustained thrombocytopenia compared to those without.

**Conclusion:**

Early platelet dynamics during SAB have prognostic significance and represent an early window for potential platelet-directed therapeutic interventions to improve outcome.

## Introduction

*Staphylococcus aureus* is a leading cause of sepsis and mortality in the United States [1]. We have previously shown that despite receiving at least three days of anti-staphylococcal therapy with apparent in vitro activity, persistent growth of *S. aureus* in the blood occurs in one-third of patients with *S. aureus* bacteremia (SAB) [2]. Additionally, mortality risk is associated with a 16% increase for each continued day with SAB [3]. Recent evidence suggests that platelets play key roles in inflammation and infection in addition to thrombosis and hemostasis [4]. Platelet aggregates in the liver entrap *S. aureus* to facilitate pathogen clearance from the bloodstream [5, 6]. Upon direct interaction with bacteria or their toxins, platelets become activated and secrete microbicidal proteins that can directly kill bacteria, mediate recruitment of other immune cells by chemotaxis, and enhance macrophage activities [6, 7]. Specifically, *S. aureus* secretes alpha-toxin, which is a major virulence factor that mediates pathogenic functions in sepsis by interacting with the widely expressed host cell receptor, a disintegrin and metalloproteinase domain-containing protein 10 (ADAM10), on platelets and leukocytes. Thus, binding of *S. aureus* alpha-toxin to host cells contributes to microvascular and organ dysfunction, which results from disruption of endothelial barriers, stimulation of interleukin-1β secretion by immune cells, and platelet aggregation and consumption [5, 7]. Importantly, others have shown in an experimental model of *S. aureus* sepsis that platelet recruitment and aggregation occur immediately following infection, but alpha-toxin induces a second harmful phase of aberrant platelet aggregation in the liver that results in thrombocytopenia and compromised bacterial clearance from the blood [8].

Thrombocytopenia, typically reported as a single measurement in several studies, has been shown to be an independent predictor of mortality in critically ill patients, specifically among patients with bloodstream infection [9–11]. However, platelet dynamics during the course of SAB and their impact on patient outcome are not clear. Another study has reported a link between thrombocytopenia and a dysregulated host cytokine response; however, the causative pathogens were not reported [12]. Here, we aimed to determine the impact of platelet dynamics early during the course of SAB on patient outcome. We hypothesize that early platelet count changes during the course of SAB are linked to microbial persistence and 30-day mortality.

## Methods

### Study population

This study was conducted at two university-affiliated institutions in Los Angeles, California (USA). The study protocol was approved with a waiver of informed consent by Institutional Review Boards (Advarra IRB and USC IRB) at the respective sites. Adult patients with SAB hospitalized between July 1, 2012 and November 30, 2020 were screened. The exclusion criteria were as follows: age < 18 years, receipt of < 48 h of active anti-staphylococcal therapy, delayed initiation of active anti-staphylococcal therapy for > 48 h following SAB onset, and polymicrobial bacteremia.

### Data collection

Electronic medical records were retrospectively reviewed to obtain pertinent demographic data, including age, sex, and comorbid conditions. Antibiotic management, receipt of antiplatelet agents, chemotherapy, and other immunosuppressive drugs were documented. Laboratory and microbiologic data included vital signs, complete blood cell count, comprehensive metabolic panel, culture, and susceptibility. Daily platelet counts during the initial 7 days of SAB were recorded. Extracted data were recorded in REDCap, a HIPAA-compliant secured electronic database hosted at the University of Southern California [13].

### Study definitions

Platelet count was classified as normal (NP, ≥ 150 × 10^9^/L) or thrombocytopenia (TC, < 150 × 10^9^/L), and the degree of thrombocytopenia was classified as mild (mild TC, 100–149 × 10^9^/L) or moderate-to-severe (MS TC, < 100 × 10^9^/L) [12]. The sources of bacteremia were grouped relative to mortality risk as described in the study by Soriano et al. [14] as follows: low (< 10%), intermediate (10–20%), and high (> 20%). Low-risk sources of infection were intravenous catheters, urinary tract infection, ear-nose-larynx, gynecologic, and several manipulation-related sources. Intermediate-risk sources were osteoarticular, soft-tissue, and unknown sources. High-risk sources were endovascular, lower respiratory tract, intra-abdominal, and central nervous system foci [14]. Outcome endpoints were mortality (primary) and persistent bacteremia (secondary). Mortality was assessed at 30 days after SAB onset. Day 1 or SAB onset was defined as the day of the first positive blood culture with *S. aureus,* and Day 4 was defined as 72 h thereafter [15]. Persistent bacteremia was defined by positive blood cultures for three days or longer [3].

### Data analysis

Patients were grouped by their platelet count per the above definitions (NP, TC, mild TC, and MS TC) at onset as well as by the changing platelet count trend between Day 1 and Day 4 from onset of SAB and over the initial 7-day period. Study groups were compared for overall clinical characteristics based on platelet count at onset. Descriptive analyses were performed using independent two-sample t tests or Mann‒Whitney U tests for continuous variables and chi-square or Fisher’s exact tests for categorical variables. A *p value* < 0.05 was considered statistically significant. Hommel’s multiple testing method was used to adjust p values when comparing the clinical outcomes of patients grouped by platelet dynamics. A modified Poisson regression analysis using error variance was used to analyze platelet count on Day 4 as a continuous variable to identify the incremental risk for death with every incremental 20-unit drop in platelet count using 150 × 10^9^/L as the reference group. Multivariable regression analyses were performed to determine the association between clinical factors and the 30-day mortality outcome. The final multivariable model and submodels were derived by combining the models produced by backward and forward selections after assessing the multicollinearity among possible candidates. The common factors were controlled in the model and submodels for comparison. Hosmer and Lemeshow goodness-of-fit tests were performed in the logistic regression models. Statistical analyses were performed using GraphPad Prism version 9.1.2 (GraphPad Prism, San Diego) and SAS version 9.4 (SAS Institute).

## Results

### Patient characteristics associated with thrombocytopenia at onset of SAB

A total of 842 patients hospitalized with SAB between July 1, 2012 and November 30, 2020 were screened. Of those, 811 patients met the inclusion criteria. Overall, the majority of the patients were male (71%), and the median age was 59 years. The most common comorbidities were hypertension (50%), diabetes (43%), and renal disease (26%) (Table 1). Thrombocytopenia occurred in 29% of patients on Day 1, of whom 15% had mild and 14% had MS TC. Patients with TC on Day 1 were more likely to present with coronary artery disease (17% vs. 9%, *p* = 0.002) and renal disease (31% vs. 24%, *p* = 0.04) than those with NP. In addition, TC patients were more likely to have alcohol use disorder (19% vs. 12%*, p* = 0.01), active malignancy (15% vs. 8%, *p* = 0.001), and liver disease (28% vs. 10%, *p* < 0.0001), particularly among patients with MS TC (data not shown). The majority of the patients had acquired SAB in the community with a higher proportion in the NP group than in the TC group (85% vs. 79%, p = 0.03), but MRSA was the causative pathogen in 34% of bacteraemia cases irrespective of platelet count on Day 1 (TC vs. NP: 30% vs. 36%, *p* = 0.10). Compared to patients with NP on Day 1, those with TC on Day 1 were significantly more likely to be associated with an infection from a high-risk source (32% vs. 18%, *p* < 0.0001), to present with endocarditis as their primary source of infection (17% vs. 6%, *p* < 0.0001), and to be critically ill as reflected by the presence of septic shock (18% vs. 11%, *p* = 0.003) and a higher proportion requiring care in the intensive care unit (ICU) (43% vs. 29%, *p* = 0.0001). Notably, among patients with TC, a significantly greater number of patients with MS TC experienced septic shock compared to those with mild TC or NP (21% vs. 16% vs. 11%, *p* = 0.005) (data not shown). The most common initial anti-staphylococcal agent was a vancomycin-containing regimen (74%). Linezolid-containing regimens were used in only 5% of the overall patients. TC patients were less likely to have source control procedures performed (65% vs. 52%, *p* < 0.001) (Table 1).

**Table 1:** Demographics, clinical presentation and management grouped by platelet count at onset of *S. aureus* bacteremia

	All patients N = 811	NP (≥ 150 × 10^9^/L) n = 577	TC (< 150 × 10^9^/L) n = 234	p-value (NP vs TC)
Age, years, median (IQR)	59 (47, 70)	58 (47, 69)	59 (49, 72)	0.15
Male	577 (71)	416 (72)	161 (69)	0.35
Comorbidities	–	–	–	–
Coronary artery disease	94 (12)	54 (9)	40 (17)	0.002
Diabetes	350 (43)	259 (45)	91 (39)	0.12
Hypertension	402 (50)	287 (50)	115 (49)	0.88
Intravenous drug use	97 (12)	73 (13)	24 (10)	0.34
Alcohol use disorder	117 (14)	72 (12)	45 (19)	0.01
Active malignancy	79 (10)	44 (8)	35 (15)	0.001
Liver disease	123 (15)	57 (10)	66 (28)	< 0.0001
Cirrhosis	82 (10)	24 (4)	58 (25)	< 0.0001
Renal disease	213 (26)	140 (24)	73 (31)	0.04
Community Onset of SAB	677 (83)	492 (85)	185 (79)	0.03
MRSA as causative pathogen	277 (34)	207 (36)	70 (30)	0.10
Source risk category	–	–	–	< 0.0001
Low	166 (20)	104 (18)	62 (27)	
Intermediate	469 (58)	372 (64)	97 (41)	
High	176 (22)	101 (18)	75 (32)	
Infection type related to SAB	–	–	–	–
Endocarditis	73 (9)	34 (6)	39 (17)	< 0.0001
Pneumonia	69 (9)	46 (8)	23 (10)	0.39
Osteomyelitis	101 (12)	87 (15)	14 (6)	0.0004
Metastatic Complications^^^	176 (22)	127 (22)	49 (21)	0.72
Severity of Illness	–	–	–	–
Pitt Bacteremia Score,median (IQR)^^^	1.0 (0, 2.0)	1.0 (0, 2.0)	1.0 (0, 3.0)	0.0003
Severe sepsis^^^	363 (45)	209 (37)	154 (67)	< 0.0001
Septic shock^^^	104 (13)	61 (11)	43 (18)	0.003
ICU stay^^^	269 (33)	168 (29)	101 (43)	0.0001
Source control procedure not performed	452 (56)	300 (52)	152 (65)	< 0.001
Initial anti-staphylococcal regimen	–	–	–	–
Vancomycin-containing regimen	597 (74)	428 (74)	169 (72)	0.57
Linezolid-containing regimen	37 (5)	32 (6)	5 (2)	0.04
Time to start of effective anti-staphylococcal therapy	–	–	–	0.88
On or before day of SAB onset	567 (70)	404 (70)	163 (70)	
Within 24 h of SAB onset	220 (27)	156 (27)	64 (28)	
Within 48 h of SAB onset	21 (3)	16 (3)	5 (2)	
Concurrent antiplatelet therapy^*^	199 (25)	140 (24)	59 (25)	0.78
Aspirin	193 (24)	135 (23)	58 (25)	0.67
Clopidogrel	35 (4)	25 (4)	10 (4)	0.97

ICU: intensive care unit; IQR: interqartile range; NP: normal platelet; SAB: *S. aureus* bacteremia; TC: thrombocytopenia

Data are no. (%) unless otherwise indicated

^*^Aspirin, clopidogrel, aspirin-dipyridamole; ^data may not add up to N = 811 due to missing data

### Platelet dynamics during SAB

**Fig. 1:**
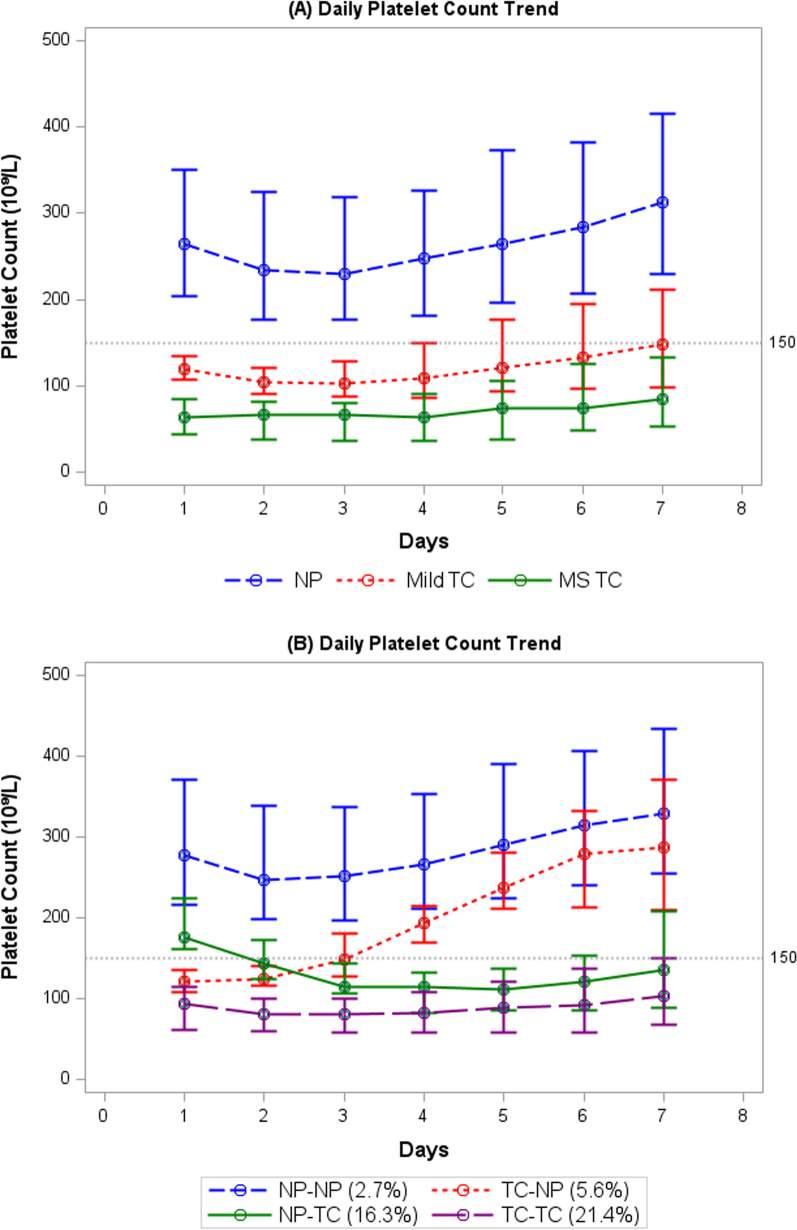
**A**, **B** Platelet trends over the first seven days of *S. aureus* bacteremia. Groups compared were: **A** Normal Platelet (NP) vs Mild Thrombocytopenia (Mild TC) vs Moderate-to-Severe Thrombocytopenia (MS TC); **B** Platelet trend for 4 groups based on platelet count on Day 1 and Day 4 and respective mortality rate (%): TC-TC (thrombocytopenia at both timepoints), 21.4%; TC-NP (Thrombocytopenia on Day 1 with recovery to normal platelet count on Day 4), 5.6%; NP-NP (normal platelet count entire course), 2.7%; NP-TC (normal platelet count on Day 1 but thrombocytopenia on Day 4), 16.3%

The trend in platelet count over the initial 7-day period following SAB onset is depicted in Fig. 1A. Regardless of the platelet count on Day 1, the platelet count decreased from SAB onset to reach a nadir by Days 2–3 in general. In the cohort with daily platelet count over the initial 7-day period for thrombocytopenia (n = 451), a change in platelet count was observed from normal to below normal levels and vice versa during the initial four days of SAB in a subset of patients. Among patients with normal platelet count on Day 1, 11% (65/577) became thrombocytopenic (NP-TC) by Day 4, whereas 14% (33/234) who were TC on Day 1 had recovery of platelet count to normal level (TC-NP) by Day 4. The platelet trend for the four groups based on platelet count on Day 1 and Day 4 with respective mortality rate is shown in Fig. 1B.

### Early trends in platelet count predict microbial persistence and mortality

Outcome analyses in relation to platelet dynamics were performed on the subset of patients (n = 752) who had platelet counts on both Day 1 and Day 4 (Table 2). Overall, persistent bacteremia and 30-day mortality occurred in 45% and 9% of this subset of patients, respectively. When grouped by platelet dynamics, persistent bacteremia occurred most frequently in the TC-TC group at 54%, followed by NP-TC (51%), TC-NP (42%), and NP-NP (41%) (Table 2). The duration of bacteremia ranged between 1 and 23 days in the present cohort. Among patients who experienced sustained TC, the duration of bacteremia was significantly prolonged by one day compared to those who had normal platelet counts throughout (median: 3 vs. 2 days, respectively, p = 0.005).

**Table 2:** Comparison of clinical outcome grouped by platelet dynamics (N = 752)

	TC-TC n = 182	NP-TC n = 65	TC-NP n = 33	NP-NP n = 472	p-value (TC-TC vs NP-TC)	p-value (TC-TC vs TC-NP)	p-value (TC-TC vs NP-NP)	p-value (NP-TC vs TC-NP)	p-value (NP-TC vs NP-NP)	p-value (TC-NP vs NP-NP)
Duration of SAB, days, median (IQR)	3.0 (1.0–5.0)	3.0 (1.0–4.0)	2.0 (1.0–5.0)	2.0 (1.0–4.0)	1.00	1.00	0.005	0.98	0.10	0.98
Persistent SAB	98 (54)	33 (51)	14 (42)	191 (41)	0.86	0.84	0.01	0.86	0.65	0.86
30-day mortality	38 (21)	12 (18)	2 (6)	13 (3)	0.68	0.16	< 0.001	0.39	< 0.001	0.52

TC-TC: thrombocytopenia on Day 1 and Day 4; NP-TC: normal platelet count on Day 1 but thrombocytopenia on Day 4; TC-NP: Thrombocytopenia on Day 1 with recovery to normal platelet count on Day 4; NP-NP: normal platelet count entire course; IQR: interquartile range; SAB: *S. aureus* bacteremia

Data are no. (%) unless otherwise indicated; N = 752 due to missing platelet counts at day 4

p-values were adjusted using Hommel’s multiple testing method

Consistent with the above trend, mortality was highest among patients in the TC-TC group (21%). Mortality risk corresponded to the degree of severity of TC on Day 4 (26%, 12%, and 3% for MS TC, mild TC, and NP, respectively, p < 0.0001) (data not shown). Compared to patients whose platelet count remained normal between Day 1 and Day 4 (NP-NP), the group that developed TC by Day 4 (NP-TC) had a sixfold greater odds for death (18% vs. 3%, *p* < 0.001). Notably, mortality was greatly reduced among patients whose platelet count recovered to within the normal range by Day 4 (TC-NP) compared to those who remained thrombocytopenic (TC-TC); however, this difference was not statistically significant after adjustment for multiple comparisons (6% vs. 21%, *p* = 0.16), and the mortality rate approached the rate of those who did not experience thrombocytopenia at any time during the course of SAB (3%).

Patients were grouped by every 20-unit decrease in platelet count on Day 4 following the onset of SAB. When platelet count on Day 4 was analyzed as a continuous variable, a 26% increased relative risk of mortality was associated with every additional 20 × 10^9^/L drop below 150 × 10^9^/L in platelet count on Day 4 (RR 1.26, CI: 1.18–1.35, *p* < 0.0001) (Table 3). Thus, platelet count on Day 4 was a determining point of platelet trajectory during the initial 7 days of SAB and portended differential mortality risk.

**Table 3:** Relative risk (95% confidence interval) of 30-day mortality by platelet count (N = 752)

Platelet count on Day 4	Total N	Mortality %	Relative risk (95% CI)	p-value
> 150	504	0.2	Reference	N/A
131–150	39	2.6	3.45 (1.20–9.88)	0.02
111–130	41	2.4	1.64 (0.39–6.92)	0.50
91–110	42	2.4	5.60 (2.42–12.97)	< 0.0001
71–90	47	2.1	8.58 (4.27–17.24)	< 0.0001
51–70	41	2.4	12.29 (6.48–23.34)	< 0.0001
< = 50	38	2.6	8.84 (4.27–18.33)	< 0.0001
Per 20-unit of platelet count			1.26 (1.18–1.35)	< 0.0001

N = 752 due to missing platelet counts at day 4

### Predictors of mortality identified by logistic regression analysis

Taking into account the changes in platelet count between Day 1 and Day 4, multivariable regression analyses were performed to identify the association between clinical variables and the outcome mortality in the subset of patients who had platelet counts on both Day 1 and Day 4. Clinical variables considered in the model selection were changes in platelet trend (grouped by platelet count changes between Day 1 and Day 4), age, sex, heart failure, renal disease, source risk of bacteremia, endocarditis, pneumonia, severe sepsis, septic shock, Pitt bacteremia score (PBS), source control procedure not performed, ICU stay, and MRSA. The significant factors strongly associated with 30-day mortality were sustained thrombocytopenia or thrombocytopenia developed by Day 4, high-risk source of infection, severe sepsis, age, ICU stay, renal disease, and source control procedure not performed (Table 4). Interestingly, different drivers for mortality were identified when submodel analyses were performed based on platelet count at onset of infection. For patients with normal platelet count at onset, significant predictors of 30-day mortality were a decrease in platelet count to below normal on Day 4, age, ICU stay, renal disease, and lack of a source control procedure. For patients with thrombocytopenia at onset, significant predictors for mortality were sustained TC on Day 4, high-risk source of infection, severe sepsis, ICU stay, and infection with MRSA.

**Table 4:** Multivariable models for 30-day mortality (n = 740) and subgroups based on platelet count trends from *S. aureus* bacteremia onset to Day 4

Variables	OR	95% CI		p-value
**Full Model: All patients (N = 740)**				
TC-TC vs NP-NP	4.42	2.12	9.21	< 0.0001
TC-NP vs NP-NP	1.88	0.36	9.84	0.46
NP-TC vs NP-NP	3.26	1.25	8.53	0.02
High risk source vs Intermediate & Low	2.42	1.29	4.55	< 0.01
Severe Sepsis	3.84	1.50	9.83	< 0.01
Age	1.02	1.00	1.04	0.02
ICU stay	6.24	2.92	13.31	< 0.0001
Source control procedure not performed	2.34	1.15	4.76	0.02
Renal disease	2.25	1.13	4.47	0.02
MRSA vs MSSA	1.36	0.73	2.54	0.33
**Submodel: Patients with normal platelet count on Day 1 (N = 529)**				
NP-TC vs NP-NP	3.57	1.34	9.49	0.01
High risk source vs Intermediate & Low	1.79	0.70	4.59	0.22
Severe Sepsis	2.17	0.69	6.80	0.18
Age	1.03	1.00	1.06	0.05
ICU stay	7.69	2.43	24.37	< 0.001
Source control procedure not performed	3.07	1.05	9.04	0.04
Renal disease	2.93	1.08	7.94	0.03
MRSA vs MSSA	0.57	0.21	1.55	0.27
**Submodel: Patients with thrombocytopenia on Day 1 (N = 211)**				
TC-TC vs TC-NP	2.06	0.40	10.66	0.39
High source risk vs Intermediate & Low	3.38	1.35	8.44	< 0.01
Severe Sepsis	10.03	1.23	81.75	0.03
Age	1.02	1.00	1.05	0.08
ICU stay	5.95	2.10	16.89	< 0.001
Source control procedure not performed	1.87	0.68	5.19	0.23
Renal disease	2.05	0.76	5.51	0.16
MRSA vs MSSA	2.64	1.10	6.34	0.03

CI: confidence interval; OR: odds ratio; TC-TC: thrombocytopenia on Day 1 and Day 4; NP-TC: normal platelet count on Day 1 but thrombocytopenia on Day 4; TC-NP: Thrombocytopenia on Day 1 with recovery to normal platelet count on Day 4; NP-NP: normal platelet count entire course; N = 740 due to missing platelet counts at day 4 and other variables with missing values

Hosmer and Lemeshow Goodness-of-Fit Test, all p-values > 0.05

## Discussion

While thrombocytopenia measured at a single timepoint has been previously shown to be a risk factor for mortality in sepsis, platelet count may fluctuate during the course of infection. The present study is the first to analyze the impact of platelet dynamics early during the course of *S. aureus* bacteremia on mortality and microbial persistence in over 800 patients. A subset of the present study cohort had changes in platelet count to either above or below the normal range within the first 4 days of SAB onset, and platelet count on Day 4 was a strong determinant of platelet trajectory and a predictor of mortality. Close to 30% of patients experienced thrombocytopenia on Day 1, and another 11% of patients developed thrombocytopenia on Day 4. However, 14% of patients with thrombocytopenia at onset had recovery of platelet count to within the normal range by Day 4. Regarding the patients who developed thrombocytopenia on Day 1, they were significantly more likely to be admitted to the ICU and more likely to have a pre-existing risk for thrombocytopenia, such as active malignancy, liver disease, and alcohol use disorder. Additionally, we and others have found that a higher proportion of patients who develop thrombocytopenia have endocarditis as well as an association between thrombocytopenia at sepsis onset and 30-day mortality [10]. Platelets have been shown to play an integral role in the thrombotic process involved in the formation of valvular vegetation. Because alpha-toxin, a key virulence factor secreted by almost all strains of *S. aureus*, binds directly to platelets and causes aberrant aggregation in the microvasculature, microthombi in the microvasculature, endothelial cell activation, and von Willebrand factor release [5, 8], it is likely that thrombocytopenia is a marker indicative of the pathogen-mediated immunothrombotic event that predisposes patients to the development of endocarditis. Notably, the present findings differed in terms of mortality from those reported by Gafter-Gvili et al. as they observed a much higher mortality rate (56%) compared to the present study (18%). Forsblom et al. showed that thrombocytopenia at Day 7 from SAB onset is associated with 90-day mortality [11], and they observed that thrombocytopenia on Day 1 is not associated with 28-day mortality.

In line with published findings of platelet count trends in the setting of an acute illness [16], we observed a biphasic trend in platelet count during the early phase of SAB; platelets decreased from the onset of bacteremia, reaching a nadir by Days 2–3 and then returning to baseline level or higher by Day 7, while a subset remained persistently thrombocytopenic. Importantly, a close examination of the platelet count trend during the initial four days of bacteremia in the present cohort indicated that platelet count on Day 4 was a strong determinant of platelet trajectory with prognostic significance regardless of platelet count on Day 1. When platelet count on Day 4 was evaluated as a continuous variable, the risk of death increased by 25% for every 20 × 10^9^/L decrease in platelet count. In particular, the risk of death in patients with normal platelet counts on Day 1 who developed thrombocytopenia by Day 4 approached that of those who had thrombocytopenia throughout the course of bacteremia (20% vs. 25%). For those with thrombocytopenia on Day 1, the risk of mortality was significantly reduced if the platelet count recovered to a normal range compared to those whose platelet count remained normal throughout the course of infection (5% vs. 3.5%). The latter observation suggested a window of opportunity for therapeutic intervention to protect platelets from destruction or to promote platelet count recovery.

Of interest, we and others have previously shown that antibiotics have differential effects on α-toxin expression [17, 18]. Selected β-lactam agents induce alpha-toxin gene expression, while some of the protein synthesis inhibitors (e.g., clindamycin, tedizolid, and linezolid) inhibit alpha-toxin protein production [17, 18]. In the present study, the majority of patients received a vancomycin-containing regimen for initial anti-staphylococcal therapy despite that only one-third of the patients had MRSA as the causative pathogen. Only 5% of the present cohort received a linezolid-containing regimen with a significantly higher proportion (6% vs. 2%) in the group with normal platelet counts compared to those with thrombocytopenia. It remains unclear whether linezolid offers a protective effect against alpha-toxin-mediated thrombocytopenia in this subgroup given the small sample size. In a study involving 100 patients with SAB (half survivors and half nonsurvivors), we have previously shown that *S. aureus* isolates exhibit varying expression levels of alpha-toxin as measured by a rabbit-based hemolytic activity assay; notably, the hemolytic activity of MRSA strains is significantly associated with the risk of thrombocytopenia and death [19]. This finding, in part, offers a biological explanation for the observation in the *present* study, in which high-risk source infection with MRSA was strongly predictive of death in the subset of patients with SAB who developed thrombocytopenia at onset. We acknowledge that the present study spanning an 8-year period likely included MRSA strains that belonged to different epidemic clones with altered virulence phenotypes, including alpha-toxin expression, which may have contributed to patient outcome. Nonetheless, these findings suggested that precision antibiotic therapy may be prescribed to patients in the future based on measures of pathogen virulence and susceptibility by harnessing the dual antimicrobial and antivirulence potential of antibiotics to improve the treatment outcome of *S. aureus* bacteremia.

This retrospective observational study had several limitations. The frequency of platelet count monitoring, antibiotic selection, and time to optimal therapy were at the discretion of providers. To our knowledge, this is the first large study to include patients with SAB with multiple platelet count measures during the course of bacteremia to assess the impact of platelet dynamics early during the course of infection on bacterial persistence and mortality. The majority of the present patients had platelet counts on at least 2 days (Day 1 and Day 4 of bacteremia), while more than half had daily platelet counts for the initial 7 days of bacteremia. For potential confounding factors associated with the risk of mortality, the final analyses were adjusted to include factors, such as MRSA, renal disease, and severe sepsis, in the full model and submodels. In the present study, we observed a consistent association between changes in platelet count from Day 1 to Day 4 and mortality in all analyses. Additionally, only a small subset of patients in the study cohort received antiplatelet agents, which precluded meaningful analysis of the effect of antiplatelet agents on platelet dynamics and outcome of SAB.

Taken together, the present findings suggested the following implications for practice: 1) daily monitoring of platelet count during the initial four days of infection in patients with SAB, particularly in those infected with MRSA; and 2) cautious use of concomitant medications with the potential to cause thrombocytopenia depending on their mechanism affecting platelets. Importantly, the present findings lend further support for performing follow-up studies to determine the feasibility of measuring the *S. aureus* alpha-toxin phenotype as part of the routine workflow in clinical microbiology as additional information to guide treatment selection as well as to assess the clinical impact of administering adjunctive alpha-toxin inhibitors or platelet-directed agents (e.g., clindamycin, ticagrelor, and oseltamivir) to protect against alpha-toxin-mediated platelet injury or depletion [20, 21] to improve the outcome of SAB.

## Data Availability

All data generated or analyzed during this study are included in this published article.
